# Advancing Cardiac Amyloidosis Care Through Insights from Cardiopulmonary Exercise Testing

**DOI:** 10.3390/jcm13237285

**Published:** 2024-11-29

**Authors:** Pietro Pugliatti, Giancarlo Trimarchi, Federico Barocelli, Fausto Pizzino, Francesco Di Spigno, Andrea Tedeschi, Maurizio Cusmà Piccione, Pierangela Irrera, Daniela Aschieri, Giampaolo Niccoli, Umberto Paradossi, Gianluca Di Bella

**Affiliations:** 1 Department of Clinical and Experimental Medicine, University of Messina, 98100 Messina, Italy; pietropugliatti@tiscali.it (P.P.); maurizio.cusmapiccione@polime.it (M.C.P.); pierangela.irrera@unime.it (P.I.); gianluca.dibella@unime.it (G.D.B.); 2 Interdisciplinary Center for Health Sciences, Scuola Superiore Sant’Anna, 56127 Pisa, Italy; 3 Cardiology Division, Parma University Hospital, 43126 Parma, Italy; federico.barocelli@gmail.com (F.B.); giampaolo.niccoli@unipr.it (G.N.); 4 Fondazione Toscana G. Monasterio, Ospedale del Cuore G, Pasquinucci, 54100 Massa, Italy; fpizzino@ftgm.it (F.P.); uparadossi@ftgm.it (U.P.); 5 Cardiology Unit of Emergency Department, Guglielmo da Saliceto Hospital, 29121 Piacenza, Italy; francesco.dispigno@yahoo.com (F.D.S.); andrea.tedeschimd@gmail.com (A.T.); d.aschieri@ausl.pc.it (D.A.); 6 Department of Medicine and Surgery, University of Parma, 43121 Parma, Italy

**Keywords:** transthyretin amyloid cardiomyopathy, cardiopulmonary exercise testing, heart failure, peak oxygen uptake

## Abstract

Cardiac amyloidosis, encompassing both transthyretin (ATTR) and light-chain (AL) types, poses considerable challenges in patient management due to its intricate pathophysiology and progressive course. This narrative review elucidates the pivotal role of cardiopulmonary exercise testing (CPET) in the assessment of these patients. CPET is essential for evaluating disease progression by measuring cardio-respiratory performance and providing prognostic insights. This functional test is crucial not only for tracking the disease trajectory, but also for assessing the effectiveness of disease-modifying therapies. Moreover, CPET facilitates the customization of therapeutic strategies based on individual patient performance, enhancing personalized care. By objectively measuring parameters such as peak oxygen uptake (VO_2_ peak), ventilatory efficiency, and exercise capacity, clinicians can gain a deeper understanding of the degree of functional impairment and make informed decisions regarding treatment initiation, adjustment, and anticipated outcomes. This review emphasizes the importance of CPET in advancing personalized medicine approaches, ultimately striving to improve the quality of life and clinical outcomes for patients with cardiac amyloidosis.

## 1. Introduction

Amyloidosis is a systemic infiltrative disease typically involving several tissues and organs, including the heart, nervous system, kidneys, skin, gut, and liver. It is characterized by structural and functional alterations caused by the deposition of amyloid in the tissues, an amorphous and insoluble proteinaceous material composed of fibrils with a typical β-sheet secondary structure, which makes it very difficult to degrade by proteolytic cellular enzymes, favoring accumulation in the interstitial space of the target organs [1]. Heart involvement in amyloidosis is usually associated with poor prognosis and progression to heart failure. Several types of amyloidosis can show cardiac involvement; however, transthyretin amyloidosis (ATTR) and light-chain amyloidosis (AL) are the most frequently associated with cardiac amyloidosis (CA) [2].

Transthyretin is a circulating tetrameric protein produced by the liver that acts as a carrier for thyroxine and retinol. The presence of genetically induced alterations in the amino acid sequence can trigger the amyloidogenic process. These genetic alterations can be either hereditary (ATTR variant—ATTRv) or acquired (ATTR wild type—ATTRwt), with the latter form, previously referred to as ‘senile’, usually showing a high incidence and prevalence in elderly individuals. AL amyloidosis is typically due to the misfolding of the light-chains of immunoglobulins. In this case, the amyloidogenic process is usually secondary to an excessive production of altered monoclonal immunoglobulins by a plasma cell clone in the context of multiple myeloma (MM). AL amyloidosis is usually characterized by a more significant direct cytotoxic effect of the amyloid fibrils, leading to a more rapid disease progression and worse prognosis compared to ATTR, which typically presents a more indolent behavior.

Both types are characterized by myocardial pseudo-hypertrophy and diastolic dysfunction, while systolic dysfunction appears only in the most advanced stages of the disease. As a consequence, CA represents one of the most paradigmatic examples of heart failure with preserved ejection fraction (HFpEF) [3]. Unlike the “mildly reduced” and “reduced” EF types of HF, where EF represents an immediate and reliable parameter, prognostic assessment is more challenging in patients with HFpEF. In this setting, the cardiopulmonary exercise test (CPET) has recently demonstrated its ability to provide pivotal diagnostic and prognostic information in addition to other techniques, including advanced imaging methods, electrocardiography (ECG), and biomarkers [4].

CPET is a unique method able to analyze the functional ability of the cardiocirculatory, pulmonary, and muscular systems during standardized physical exercise. CPET is typically performed with the patient wearing a mask equipped with sensors for gas exchange. Exercise is usually performed on a treadmill or on a cycle ergometer with a predefined protocol [5]. CPET provides the opportunity to monitor several parameters simultaneously, including heart rate (HR), oxygen uptake (VO_2_), carbon dioxide output (VCO_2_), and minute pulmonary ventilation (VE). Many other parameters are derived from gas flow and gas concentrations [oxygen (O_2_) and carbon dioxide (CO_2_)] during exercise. The most important and widely used parameter is peak VO_2_ (VO_2_max). According to the Fick formula, VO_2_ max = (HR × stroke volume) × arterial-venous delta O_2_. A reduced VO_2_max below 85% of the predicted value represents a negative prognostic marker in many cardiopulmonary diseases, including HF, pulmonary hypertension (PH), cardiomyopathies, and chronic obstructive pulmonary disease (COPD) [6].

**Figure 1:**
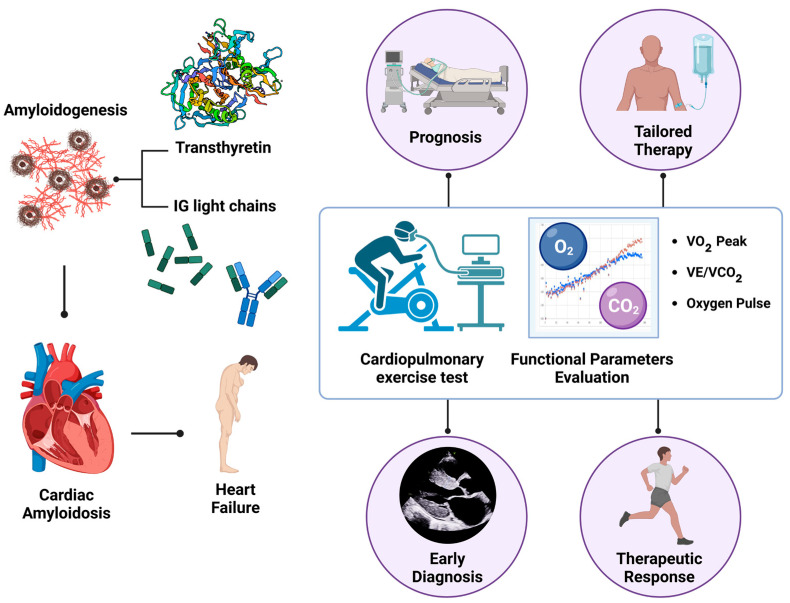
Graphical abstract summarizing the role of the cardiopulmonary exercise test (CPET) in cardiac amyloidosis (CA).

This narrative review aims to elucidate the critical role of CPET in the assessment of patients with HF secondary to CA. The unequivocal advantages of CPET, including its ability to provide comprehensive insights into a patient’s functional capacity and cardiopulmonary response to exertion, make it an invaluable tool in this specific context. By investigating the strengths of CPET, this review seeks to underscore its contributions to more tailored and effective treatment strategies within personalized medicine frameworks. It highlights how CPET facilitates not only the stratification of patients according to their exercise tolerance and symptomatology, but also the optimization of therapeutic interventions aimed at enhancing overall health outcomes. By fostering a deeper understanding of the capabilities and limitations of CPET, this narrative review ultimately aspires to pave the way for improved quality of life and clinical outcomes for patients grappling with the complexities of cardiac amyloidosis, thereby emphasizing the ongoing need for innovative approaches in cardiovascular medicine (Figure 1).

## 2. Cardiac Amyloidosis: From Diagnosis to Risk Stratification

The diagnosis of CA begins with clinical suspicion of the disease, which should be raised by several clinical, electrocardiographic, laboratorial, and imaging signs of the disease. Regarding clinical features, these could be slightly different according to the type of the disease [7,8]. In AL amyloidosis, the myocardium is the most frequently affected organ, with heart failure signs dominating the clinical scene, including dyspnea, shortness of breath, asthenia, and ankle swelling. The second most frequent clinical manifestation is nephropathy, presenting with nephrotic syndrome, followed by liver disease with ascites and neuropathy. Macroglossia and periorbital purpura are typical of this form of cardiac amyloidosis [9]. ATTRv’s main clinical manifestation is peripheral neuropathy followed by cardiac involvement [10]. The mutations most frequently associated with heart involvement are Val122Ile, Ile68Leu, Leu111Met, and Thr60Ala [11,12].

Regarding ATTRwt amyloidosis, symptoms related to cardiac involvement are the most frequent. Carpal tunnel syndrome is frequent, and bilateral involvement has been associated with a 31-fold increased risk of ATTR CA. Brachial biceps tendon rupture, trigger finger, rotator cuff disease, lumbar spinal stenosis, and large joints osteoarthritis are common [9]. Often, the first suspicion of the disease rises from incidental findings of increased cardiac wall thickness. Transthoracic echocardiography (TTE) is the most commonly used method for revealing increased cardiac wall thickness because of its reliability, availability, and repeatability [13,14]. In recent years, several echocardiographic “red flags” have been identified, including the following: mild pericardial effusion; increased thickness of the interatrial septum, atrioventricular valves, and Eustachian valve; atrial enlargement; diastolic dysfunction; and a reduction in global longitudinal strain (GLS) that is more pronounced in the basal and middle segments, with apical sparing [15,16,17]. Furthermore, employing advanced strain-derived tools, such as Myocardial Work analysis, can aid in the differential diagnosis of hypertrophic phenotypes [18,19,20].

Regarding ECG, atrial fibrillation is common as well as AV blocks [21,22]. Low ECG voltages can be present in peripheral leads, even though it is most common to find a discrepancy between echocardiography-detected wall thickness and voltages (i.e., greatly increased wall thickening with normal voltages) [23]. A “pseudonecrosis pattern” could be present when a reduction in voltage “deletes” the small r waves with apparent Q waves.

Laboratory-derived biomarkers are useful in diagnosis and prognosis; these include high sensitive troponin, particularly troponin T, and NT-pro-BNP. Although the alteration of biomarkers is not specific to CA, their alteration represents a further clue for diagnosis; moreover, they are a significant prognostic indicator, and are useful in monitoring therapeutic response.

Cardiac magnetic resonance (CMR) has emerged in recent years as a very reliable instrument in the diagnosis of CA [7,24,25]. The use of a gadolinium-based contrast means acquiring T1-weighted gradient echo sequences after 5–10 min [late gadolinium enhancement (LGE)], which allows for the identification of the typical CA pattern characterized by diffuse contrast enhancement of the myocardium with relatively poor enhancement of the blood pool (low contrast between the blood pool and myocardium) [26].

The development of quantitative T1 analysis has given the opportunity to better characterize amyloid deposition, which typically increases the native T1 values [27,28], while the use of contrast permits the indirect quantification of extracellular volume (ECV), which is typically increased in CA (the normal value is 28%, while values > 40% are very likely for CA) [29].

Once the suspicion of CA has been raised according to clinical, laboratorial, echocardiographic, CMR, and ECG red flags, the first step is to perform a laboratory test to exclude AL-CA, particularly a serum free light-chain assay and serum and urine immunofixation electrophoresis [30]. The finding of a monoclonal free light-chain component necessitates the initiation of the search for and treatment of the associated hematological dyscrasia; a myocardial biopsy and cardiac magnetic resonance imaging can confirm myocardial involvement.

In the absence of monoclonal processes, the key diagnostic method is myocardial scintigraphy, performed with bone-avid tracers such as Technetium-99m and, more recently, 99mTc-3,3-diphosphono-1,2-propanodicarboxylic acid [31]. Cardiac radiotracer uptake is semi-quantitatively graded, comparing myocardial uptake with rib uptake in a four-grade score, where 0 represents no myocardial uptake and normal rib uptake, and 3 represents that myocardial uptake is higher than rib uptake (Perugini score) [32]. Grades 2–3 show a very high specificity close to 100% for ATTR CA; notably, about 20% of AL amyloidosis can show a significant myocardial uptake [33].

Interestingly, some ATTRv mutations may not show significant uptake in cardiac scintigraphy. However, once AL-CA has been excluded through laboratory tests, the finding of Perugini 2–3 is indicative of ATTR amyloidosis. Subsequent genetic tests can identify genetic variants, and if genetic tests are negative, a diagnosis of ATTRwt-CA can be made. In the case of Perugini 0, CA can be excluded. It is more difficult to manage cases of Perugini 1; in these cases, only a myocardial biopsy can assess the presence of CA. Thanks to the development of alternative non-invasive diagnostic methods, the role of endomyocardial biopsy is now limited to controversial cases and to confirm organ infiltration; moreover, it is the most accurate technique for discriminating between the different types of amyloid [34]. Histological and histochemical techniques, including Congo red staining, allow for the identification of amyloid deposits, giving the characteristic apple-green birefringence under polarized light. The identification of amyloid types relies on immunohistochemistry and mass spectrometry techniques [35,36].

Risk stratification in amyloidosis is essential for the correct management of therapy. Currently, the majority of scoring methods, including the most-used Mayo clinic model, are based on biomarkers, and particularly on NT-pro-BNP and Troponin T dosages [37,38,39]. Among imaging methods, GLS and atrial strain derived by echocardiography have shown important prognostic insights [40,41,42]; in addition, parameters derived by CMR, including GLS and extracellular volume estimation, have demonstrated robust prognostic roles [43,44].

## 3. Cardiopulmonary Exercise Testing in Evaluating Heart Failure

Heart failure (HF) is a condition characterized by the heart’s inability to pump blood adequately to meet the body’s metabolic demands, or by it only being able to do so by operating at elevated filling pressures. Reduced functional capacity is one of the most significant clinical signs in patients with this prevalent condition, especially considering its prognostic impact [45]. The cardiopulmonary exercise test (CPET) is the gold standard for functional assessment in HF patients. This test provides a comprehensive, non-invasive evaluation of the patient’s exercise capacity, allowing for the objective measurement of functional limitations and exercise response [46,47]. By assessing numerous parameters, CPET offers a global assessment of multiple organ functions (cardiac, vascular, pulmonary, and muscular), offering the possibility of identifying the primary cause of functional limitation in these patients, which might not be apparent or identifiable at rest [48]. This information is also crucial for distinguishing the various stages of the disease and for guiding the therapeutic approach for these patients [49,50,51]. The interpretation of CPET requires a deep understanding of exercise physiology and the parameters derived from the test that provide information about the functions being evaluated. The testing can be performed using either a constant ramp or incremental steps. The choice of ramp should be based on the patient’s estimated functional capacity, considering factors such as age, anthropometric characteristics, and expected performance based on clinical features [52]. Oxygen uptake (VO_2_) represents the most studied and reliable parameter in assessing the functional capacity of patients with HF, where peak VO_2_ is connected to peak cardiac output (CO) and the perfusion of exercising muscles. During the exercise, HF patients often cannot increase their CO, leading to reduced muscle perfusion and a subsequent shift to anaerobic metabolism, resulting in muscle fatigue. Consequently, these patients often do not achieve a true VO_2_ max (predicted for sex, age, and body weight), and the VO_2_ measured at the end of exercise is referred to as peak Vo_2_. This parameter serves as an important prognostic predictor. In particular, identifying a VO_2_ peak < 12 mL/kg/min is one of the consideration criteria for listing for heart transplantation in advanced HF [53]. The prognostic role of a reduced peak VO_2_ has also been confirmed in the population of patients with HF with preserved ejection fraction (HFpEF) [54], where it remains a sensitive but not specific parameter in discriminating functional limitation from other causes of dyspnea, being a specific indicator only at very high or low values [55]. In this specific patient setting, often burdened by pulmonary comorbidities, the assessment of ventilatory reserve, evaluated as the difference between maximum voluntary ventilation (MVV) and peak exercise ventilation (VE) (<15% reserve indicating a mechanical ventilatory limitation), may facilitate a more accurate differential diagnosis [56]. In the evaluation of the cardiogenic limitation characteristics of HF patients, other extremely important parameters are VO_2_ work and oxygen pulse (VO_2_/heart rate). Vo_2_ work describes the relationship between VO_2_ and the amount of work performed (watt), and it is essential in assessing aerobic efficiency and cardiovascular function during exercise. The O_2_ pulse is derived from the ratio of oxygen uptake to heart rate, providing an indirect measure of stroke volume and the efficiency of body oxygen utilization during exercise. Due to cardiac inefficiency, these parameters are often reduced in both HF with reduced ejection fraction (HFrEF) and HFpEF patients [47]. Interestingly, peak O_2_ pulse has been reported as a good indicator of the potential for improving peak VO_2_ through exercise training in HFpEF patients [57]. Another essential parameter in the evaluation of HF patients is VE/carbon dioxide production (VCO_2_), which represents the matching of ventilation and perfusion within the pulmonary system, with a ratio < 30 L/min considered normal [58]. An elevation of the VE/VCO_2_ slope indicates that a higher minute ventilation is necessary to eliminate CO_2_, thus indicating system inefficiency. A pathological increase in the VE/VCO_2_ ratio is a common finding in patients with HFrEF and HFpEF, in which it has been demonstrated to be a significant predictor of major adverse cardiovascular events (MACE) [59]. In a recent study, Nadrud et al. demonstrated that the use of a VO_2_ and VE/VCO_2_ slope provides additional predictive value over LVEF and clinical characteristics in predicting outcomes in patients with HFpEF, for which the ability to discriminate risk was found to be greater compared to patients with HFrEF [49]. A significant increase in the VE/VCO_2_ slope, in the absence of an alternative explanation, should always prompt consideration of increased pulmonary vascular resistance (PVR), as this is an important parameter in the diagnostic and prognostic assessment of pulmonary arterial hypertension [60]. Numerous parameters are ultimately designed to explore the ventilatory function of the patient, which is often compromised in patients with multiple comorbidities, such as those with HF. Therefore, their interpretation is important for differentiating the cause of the functional limitation [17].

## 4. Cardiopulmonary Exercise Testing in Cardiac Amyloidosis: Specific Features and Insights

Cardiopulmonary exercise testing with gas exchange measurement not only determines peak oxygen uptake (VO_2_ peak), but also uncovers mechanisms behind exercise limitations, such as ventilatory, cardiovascular, and metabolic/muscular constraints. The VO_2_ peak is defined as the maximum efficiency with which the cardiovascular system can deliver oxygen to the exercising skeletal muscles, and the muscles’ ability to extract oxygen from the blood [61]. Consequently, exercise tolerance is dependent on the performance of the cardiovascular system, the efficiency of pulmonary gas exchange, and the metabolic function of the skeletal muscles. By analyzing these factors, valuable insights into the individual’s exercise capacity and limitations can be obtained, which are helpful in tailoring specific interventions for improving overall physical performance [62].

These considerations highlight the extent to which CPET provides a more comprehensive evaluation of the body’s response to physical exertion. While other non-invasive diagnostic methods, such as exercise echocardiography, can also reveal exercise intolerance and the inability to increase stroke volume, CPET offers a more complete assessment of both cardiovascular and respiratory responses during exercise [63].

**Figure 2:**
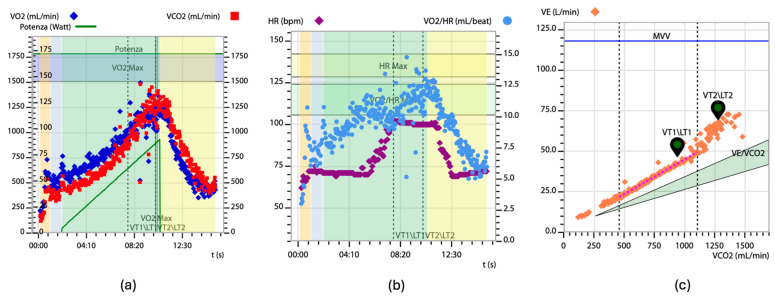
Wasserman Graphs. A 79-year-old male with transthyretin wild-type cardiac amyloidosis. Reduced peak VO_2_ of 13.1 mL/kg/min, corresponding to 67% of the predicted value (**a**). Heart rate plateau at peak exercise, suggestive of chronotropic incompetence (**b**). Increased slope of VE/VCO_2_ curve (**c**). Abbreviations: VO_2_—oxygen uptake, VCO_2_—carbon dioxide production, VE—pulmonary ventilation, HR—heart rate.

During CPET, the key parameters estimated include oxygen uptake (VO_2_), pulmonary carbon dioxide elimination (VCO_2_), minute ventilation (VE), heart rate, and systemic arterial pressure rise. Exercise intolerance in patients with CA was first reported in cases of exertional syncope [64]. Severely reduced aerobic capacity (VO_2_ peak) in CA patients has been demonstrated using the 6 min walk test and CPET [65,66,67,68]. The major CPET characteristics of patients with CA are a reduced VO_2_ peak (indicated by low absolute peak values and an anaerobic threshold VO_2_), an increased VE/VCO_2_ slope (Figure 2), and episodes of oscillatory ventilation (EOV). These patients often exhibit hyperventilation with rapid, shallow breathing during exercise, despite a normal ventilatory reserve, indicating no ventilatory limitation. Additionally, many CA patients experience chronotropic incompetence, defined as inadequate cardio-acceleration (typically less than 80–85%) at peak exercise [69,70].

In most patients with CA, the typical increase in stroke volume during exercise is absent, making heart rate elevation the crucial factor for rising cardiac output. Hence, chronotropic incompetence can significantly limit exercise capacity in these patients. However, before diagnosing chronotropic incompetence, it is essential to evaluate the level of effort achieved and the reasons for stopping the effort [71]. Low exercise intensity results in minimal metabolic stress and an inadequate heart rate increase, which does not indicate true chronotropic incompetence. Chronotropic incompetence can be objectively shown by calculating the slope for the relationship between heart rate rise and VO_2_ increase during exercise. Using this metabolic–chronotropic relationship method, no evidence of chronotropic incompetence has been found in patients with ATTR CA [72].

Despite the 6 min walking test (6 MWT) being a valuable tool for refining risk stratification and assessing the effectiveness of disease-modifying therapies, it has significant limitations, particularly in specific patient cohorts [73]. More specifically, the results of the 6 MWT can be compromised in patients with wild-type transthyretin amyloidosis (ATTRwt) who, due to effective therapies, reach advanced ages (>80 years) and consequently suffer from joint issues that impair their mobility. Similarly, in patients with hereditary transthyretin amyloidosis (vATTR), especially in mixed forms with both neuropathic and cardiac involvement, it is challenging to distinguish walking limitations caused by neuropathy from those caused by cardiac involvement [74]. In this context, CPET could play an increasing role in optimally assessing the cardiopulmonary performance of patients affected by cardiac amyloidosis.

## 5. Predictive Significance of VO_2_ Peak in Patients with Cardiac Amyloidosis

VO_2_ peak, or peak oxygen uptake, is the highest rate at which oxygen can be taken up and utilized by the body during maximal exercise. It is measured in milliliters of oxygen per kilogram of body weight per minute (ml/kg/min). According to Fick’s principle, O_2_ is equal to the product of the cardiac output and arteriovenous oxygen difference. Cardiac output is the product of heart rate and stroke volume. By knowing the VO_2_ peak and the difference in oxygen content between the arterial and venous blood, stroke volume can be estimated. The VO_2_ peak typically increases linearly with the work rate until it plateaus at maximum effort. In healthy individuals, the VO_2_ peak should be around 80% of the predicted value based on age, height, and gender. In heart failure patients, the heart cannot deliver enough oxygen-rich blood to the exercising muscles, limiting exercise capacity. This results in significantly lower VO_2_ peak values compared to healthy individuals [75].

In CA patients, there is often a deficiency in contractile reserve, leading to an inadequate improvement in stroke volume during exercise despite increased oxygen consumption (VO_2_). This deficiency is attributed to inefficient myocardial oxidative metabolism, resulting in elevated myocardial oxygen consumption without a proportional increase in stroke work. This contributes to an impaired exercise capacity and poorer clinical outcomes [76,77]. Compensatory mechanisms include increased oxygen consumption by the heart to maintain adequate cardiac output, despite perfusion abnormalities and mitochondrial dysfunction caused by amyloid deposition [66]. Patients with ATTR typically have a small left ventricular cavity and increased myocardial stiffness. These structural changes impair the normal increase in stroke volume during exercise, necessitating higher heart rates to compensate for reduced stroke volume [78].

Together, these factors underline the decline in peak VO_2_ observed in patients with ATTR, reflecting the complex interactions among structural, metabolic, and functional changes in cardiac physiology associated with amyloidosis [79]. In a study by Badr Eslam et al., a baseline VO_2_ peak greater than 14 mL/kg/min was linked to a lower risk of death or HF rehospitalization before starting tafamidis treatment. 54% of patients were able to achieve disease stabilization, while 46% showed disease progression. Patients with a stable or improved peak VO_2_ under therapy showed a more pronounced improvement in physical performance. Additionally, the mean predicted peak VO_2_, the peak VO_2_, and the % predicted heart rate significantly increased after 9 months of therapy [80]. These findings contradict the ATTR-ACT trial, which demonstrated a decline in 6 min walk test results. This discrepancy is likely due to the fact that the patients in the trial had a worse baseline functional capacity, with a 6 min walk distance of 351 ± 121 m, a higher Nt-proBNP levels (2995.9 pg/mL), and being on higher doses of beta-blockers. These factors may partly explain the increase in the percentage predicted heart rate and the observed improvement in physical performance and higher workload in the group with a stable or improved peak VO_2_. It could also be speculated that the improvement in physical performance was driven by individual patients who had an exceptionally good response [81].

However, Wernart S et al. conducted a retrospective investigation into the CPET variables of patients with HFpEF and HFmrEF, comparing those with CA to those without CA. The study matched patients by age and ejection fraction. They found that the VO_2_ peak did not significantly impact hospitalization rates. This may be attributed to the small sample size, as the VO_2_ peak values were numerically lower in hospitalized CA patients. Additionally, CA patients exhibited a trend towards lower respiratory exchange ratios (RER < 1.05), suggesting that metabolic exertion was not fully achieved in this group, potentially making the VO_2_ peak an imprecise measure in these patients [82].

Briasoulis et al., in their prospective single-center study, observed that patients with AL amyloidosis and cardiac involvement had a median peak relative VO_2_ of 17.8 mL/kg/min, which progressively declined across Mayo stages and showed a significant inverse correlation with NT-proBNP levels. Peak VO_2_ was also positively correlated with global work efficiency and global work index among imaging parameters. However, no significant correlations were found between CPET and MRI results. This indicates that while cardiac MRI is the gold standard for the early detection and diagnosis of cardiac involvement in amyloidosis, parameters such as T1 and ECV do not have significant prognostic value. Moreover, peak VO_2_ was not significantly associated with the overall survival or cardiac response at one year, likely due to the low mortality rate and early stage of the disease at diagnosis [83].

Hein and colleagues conducted cardiopulmonary exercise testing (CPET) on 27 patients with various forms of systemic amyloidosis, and found that peak VO_2_ was an independent predictor of mortality in those with cardiac involvement [65]. Similarly, Nicol and colleagues demonstrated that both peak VO_2_ and circulatory power are independent predictors of mortality and heart failure hospitalization in patients with cardiac amyloidosis [70]. In a recent analysis of 41 patients with AL or transthyretin amyloidosis, Bhutani and colleagues indicated that peak VO_2_ is an indirect marker of light-chain toxicity [68]. In Cantone et al.’s meta-analysis, a low peak VO_2_ was linked to a poorer prognosis, showing an 11% increased risk of death for each 1-unit decrease in VO_2_ peak. This association is likely attributable to factors such as reduced stroke volume, chronotropic incompetence, and muscular deconditioning observed in patients with CA. These physiological factors contributed to a decreased exercise capacity and worse outcomes in this patient group [84]. CPET also offers valuable insight into the prevalence of chronotropic incompetence, which is crucial for guiding the clinical management of patients. Peak VO_2_ integrates various physiological responses, including heart rate, inotropic capability, ventilatory efficiency, and peripheral muscular function. This comprehensive approach makes peak VO_2_ a robust predictor of clinical outcomes in patients undergoing cardiopulmonary exercise testing [70].

## 6. VE/VCO_2_ Slope and Its Prognostic Value in Patients with Cardiac Amyloidosis

The minute ventilation/carbon dioxide production (VE/VCO_2_) slope reflects the amount of ventilation expended per liter of CO_2_ exhaled. At rest, this value typically ranges between 24 and 34, and remains in this range until after the ventilatory threshold, where it increases because minute ventilation rises at a faster rate than CO_2_ production [75]. The VE/VCO_2_ slope has strong prognostic value in patients with chronic heart failure, with an increased risk of mortality when the VE/VCO_2_ slope exceeds 32.8. It also provides useful information for managing CHF [85]. In patients with CA, the increase in the VE/VCO_2_ slope (attributed to autonomic dysfunction, excessive sympathoexcitation, and a high physiological dead space (VD/VT) ratio during exercise) may stem from restrictive hemodynamics, leading to an excessive elevation in left ventricular filling pressure and pulmonary artery pressure during exercise. This ventilatory response does not appear to be related to pulmonary amyloidosis involvement [70]. Amyloid disorders primarily affect small unmyelinated nerve fibers, particularly thinly myelinated Ad fibers and unmyelinated C fibers. One significant consequence of this involvement is autonomic dysfunction. Sympathoexcitation can enhance the ventilatory response of both peripheral and central chemoreflexes, leading to increased ventilation and an elevated VE/VCO_2_ slope [86]. Monfort et al. found that patients with ventilatory inefficiency showed lower peak VO_2_ levels, elevated ventilatory drive and exercise oscillatory ventilation (EOV), and prolonged post-exercise heart rate recovery [72]. In their multivariate analysis, only excess ventilation at anaerobic threshold (VE@ATVO_2_) remained independently associated with ventilatory inefficiency. They suggested that the underperfusion of ventilated lung alveoli, which leads to a ventilation–perfusion (VA/Q) ratio mismatch, could contribute to the observed increase in the VE/VCO_2_ slope in these patients. Furthermore, CA patients exhibiting ventilatory inefficiency showed a lower peak exercise end-tidal CO_2_ partial pressure (PETCO_2_) compared to those without ventilatory inefficiency [72].

Another mechanism involves the absence of an increase in tidal volume (VT) during exercise. This is due to the inability to enhance oxygen delivery to the respiratory muscles, resulting in a reduced respiratory muscle strength. Enhanced central and peripheral chemoreflexes likely contribute to this phenomenon, limiting VT to below 50–60% of the maximal lung vital capacity. As a result, a characteristic shallow breathing pattern with a high respiratory frequency ensues [71].

In the retrospective study by Wernart S et al., VE/VCO_2_ and O_2_ pulse max were associated with hospitalization in CA patients. Specifically, a VE/VCO_2_ slope of ≤34 was associated with a lower risk of death or heart failure rehospitalization before the initiation of tafamidis treatment [81]. Yunis et al. found that an increase in the VE/VCO_2_ slope was associated with clinical events in 56 patients diagnosed with transthyretin cardiac amyloidosis (TTR CA) [87].

Finally, Banydeen et al. demonstrated that the presence of a restrictive spirometry pattern was associated with an increased risk of major adverse cardiovascular events (MACE) in patients with transthyretin amyloid cardiomyopathy (ATTR-CA). This finding aligns with other studies [88,89,90] reporting that a restrictive ventilatory pattern is predictive of both all-cause mortality and cardiovascular mortality [91].

## 7. Impact of Cardiac Amyloidosis Treatment on Physical Performance: The Role of CPET Evaluation

Therapies for cardiac amyloidosis (CA) differ based on the specific amyloid type. They encompass supportive care for heart failure and treatments that block the production of amyloid precursor proteins. Particularly in the case of ATTR-CA, research has concentrated in recent decades on disease-modifying medications that work through various mechanisms, such as inhibiting the synthesis of amyloidogenic TTR, stabilizing the native TTR tetramer structure, and eliminating misfolded proteins [92,93,94,95].

In AL, the therapeutic approach aims to eliminate the specific clone of plasma cells responsible for producing an excess of light-chains [96]. Chemotherapy regimens, also considering drugs such as bortezomib and daratumumab, and autologous stem cell transplantation are potential effective therapeutic strategies [97,98,99]. The main trials in this field have primarily analyzed the complete hematologic response, but there are also studies that have demonstrated the usefulness of assessing exercise capacity with CPET in affected patients by identifying those with more advanced cardiac involvement [68,83]; therefore, these results suggest that CPET may also play a role in evaluating the response to and candidacy for specific therapies in patients with cardiac involvement [83]. Established risk stratification guides the treatment approach for AL patients [100].

Regarding TTR-CA, in recent years, several molecules have been examined, along with taking into consideration their contribution to modifying exercise tolerance in affected patients. For example, tafamidis, which binds to TTR, preventing tetramer dissociation and amyloidogenesis, has been shown to reduce all-cause mortality at 30 months, as well as the deterioration rate during the 6 min walk test, associated with improved cardiac function and the delay of myocardial amyloid progression [101,102]. Similarly, for small RNA-interfering (siRNA) molecules and antisense oligonucleotides, analysis in hereditary TTR amyloidosis cohorts has suggested a beneficial effect on quality of life [103] and functional capacity [81,104,105], particularly in patients with polyneuropathy. The main drug trials have analyzed functional capacity data among clinical endpoints [81,101,102,103,104,105]; the results suggest that these therapies may lead to improvements in the functional capacity parameters assessed by CPET.

At present, only a few studies have examined the assessment of functional capacity using CPET in patients with ATTR-CA undergoing specific therapy.

In a retrospective study of patients with wild-type transthyretin amyloidosis (wtATTR) who were treated with tafamidis, Dalia et al. found that one-third of patients experienced the composite outcome of all-cause mortality, heart transplantation, or the initiation of palliative inotropes within a year [106]. The aim was to evaluate the prognostic role of CPET, performed within 1 month of therapy. The study emphasized that the low peak VO_2_ linked to poor prognosis in these patients may be due to a reduced stroke volume resulting from a restrictive physiology. It was observed that a decrease in stroke volume over time in individuals with wtATTR was associated with worse outcomes. Additionally, the study discovered that a low peak VO_2_/heart rate (HR) ratio, which acts as a surrogate marker for stroke volume, was also significantly correlated with poorer outcomes [106]. The study demonstrates that CPET, by offering early indicators of poorer outcomes, can assist physicians in selecting patients for tafamidis treatment [106].

In a preliminary study on eight patients with TTR-CA, Nakaya et al. investigated exercise tolerance and changes in cardiopulmonary function after one year of tafamidis therapy. They reported that the patients with TTR-CA treated with tafamidis showed a reduction of 19.2% in anaerobic threshold (AT) and of 22.3% in peak oxygen uptake (peak VO_2_) after one year of follow-up. They speculated that the factors contributing to exercise intolerance in patients are diverse, including insufficient cardiac output, inadequate increase in perfusion to the exercising muscles, and skeletal muscle dysfunction caused by impaired peripheral oxygen extraction [107]. This decline is likely due to increased N-terminal pro-B-type natriuretic peptide (NT-proBNP) levels and a worsening of the New York Heart Association (NYHA) class, which collectively contribute to decreased exercise tolerance over time. Furthermore, the progression of frailty may have also significantly impacted the reduction in exercise capacity in these patients [107]. Significant limitations of this work are the small sample size and the single-center study design; the findings require further confirmation in larger populations.

**Figure 3:**
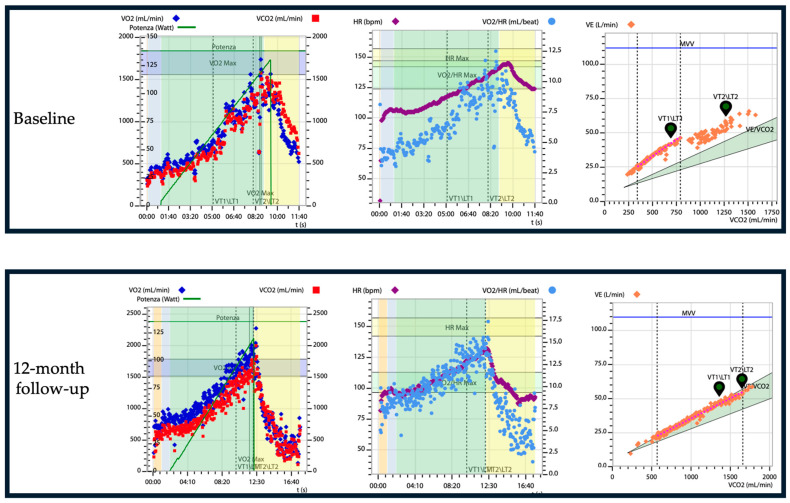
Wasserman Graphs illustrating the improvement in CPET parameters in a 64-year-old male patient with wild-type cardiac amyloidosis before and 12 months after starting tafamidis therapy. Notable improvements include an increase in VO_2_ peak (from 22 to 26) and a decrease in the slope of the VE/VCO_2_ curve. Abbreviations: VO_2_—oxygen uptake, VCO_2_—carbon dioxide production, VE—pulmonary ventilation, HR—heart rate.

A study by Badr Eslam et al. aimed to assess the effects of tafamidis and optimal background therapy on functional capacity using CPET [80]. In 54 patients who received tafamidis and underwent repeated CPET testing (follow-up CPET at 9 ± 3 months), a significant improvement in physical performance (*p* = 0.002) was observed at the follow-up. The cohort of 54 patients receiving tafamidis with baseline and follow-up CPET were divided into three groups based on their ventilatory responses: the first two groups (29 patients) were considered in a single group as the stable (peak VO_2_-change of ≤0 mL/kg/min and ≤1.0 mL/kg/min) or improved peak VO_2_ group (peak VO_2_-change of ≥1.0 mL/kg/min); in the third group (25 patients), there were patients with a decline in peak VO_2_ (peak VO_2_-change of <0 mL/kg/min) [80]. When comparing pre- and post-treatment outcomes, 29 patients (54%) demonstrated increases in percentage predicted peak VO_2_ (*p* < 0.0001), improvements in peak VO_2_ (*p* < 0.0001), and enhanced physical performance at follow-up (*p* < 0.0001). The patients who maintained or improved their peak VO_2_ had less advanced heart disease at baseline (*p* = 0.046) [80]. Compared to Nakaya et al., in the study by Badr Eslam et al., patients with ATTR-CM who had a better initial exercise tolerance and reduced β-blocker doses showed an improvement in their exercise capacity [80]. Additionally, most studies on tafamidis have demonstrated a decrease in NT-proBNP levels following its administration. Moreover, Badr Eslam et al. showed that a baseline peak VO_2_ greater than 14 mL/kg/min and a VE/VCO_2_ slope of 34 or less were linked to a reduced risk of death or hospitalization due to heart failure (*p* = 0.009 and *p* = 0.02, respectively) prior to initiating tafamidis treatment. In this study, 54% of patients who underwent repeated testing showed significant improvement in exercise capacity, as measured by CPET [80]. Those who maintained or improved their peak VO_2_ during therapy experienced a more notable enhancement in physical performance [80]. Despite these results, in the ATTR-ACT trial, a decline in the 6 min walk test performance was observed, showing that tafamidis reduced the decrease in the distance walked during the test compared to the placebo (75.68 m [standard error, ±9.24; *p* < 0.001]), with significant differences starting at the sixth month of treatment [102]. The discrepancy between the improvement in physical performance measured by CPET in the cohort of Badr Eslam et al.’s study, and the decline in the 6 min walk test results in the ATTR-ACT trial, was attributed by the authors to the fact that the subgroup of patients who completed the follow-up examination in the first study (54 patients) had a better baseline functional capacity (6 min walk distance: 420 ± 105 m) compared to the tafamidis cohort of the ATTR-ACT trial (6 min walk distance: 351 ± 121 m) [80]. Additionally, in the study by Badr Eslam et al., the patients had lower baseline NT-proBNP levels compared to the ATTR-ACT tafamidis cohort [80]. This suggests that in patients with a less advanced disease than those in the ATTR-ACT study, tafamidis might not only halt the decline in exercise capacity, but even promote its improvement. However, these findings could also be due to optimized background therapy, including reduced beta-blocker usage. It could be speculated, furthermore, that the improvement in physical performance might be driven by individuals with exceptionally good responses [80]. Figure 3 presents the case of a patient treated with tafamidis, who underwent cardiopulmonary tests at baseline and 12 months after treatment initiation.

Recent research has substantiated the efficacy and tolerability of SGLT2 inhibitors (SGLT2i) in a multicenter study led by Porcari et al., among patients diagnosed with ATTR-CA [108]. This class of medications has been associated with favorable outcomes, notably improvements in HF symptoms, renal function, and a decreased dependence on diuretic agents over time. Furthermore, SGLT2i therapy has been linked to a significant reduction in the risk of HF hospitalization, as well as lower rates of both cardiovascular and all-cause mortality, independent of the patient’s ejection fraction [108].

However, it is important to note that the study did not incorporate functional data derived from CPET within this specific patient population. This oversight highlights the necessity for future research on integrating CPET methodologies to accurately assess the impact of gliflozins on cardiopulmonary performance in patients suffering from ATTR-CA, which would contribute significantly to the understanding of nuanced physiological responses associated with this therapeutic intervention.

## 8. Future Perspectives and Clinical Applications of CPET in Cardiac Amyloidosis

In patients with amyloidosis, cardiac involvement represents one of the major negative prognostic factors, as deaths are predominantly due to heart failure or amyloidosis-associated arrhythmias. Diagnostic strategies for symptomatic cardiac amyloidosis (CA), characterized by typical morphological features, have been well described and defined, aided by recognized “red flags”. However, diagnosing subclinical CA and the early stages of the disease remains challenging and limited. Recent clinical studies have demonstrated the existence of effective drugs that suppress or delay CA progression, especially when initiated in the early stages of the disease [95]. Therefore, early detection of non-clinically overt forms of the disease is pivotal to initiating appropriate therapy. One of the current clinical objectives is to acquire tools that enable early diagnosis and provide a comprehensive assessment of response to specific therapies, moving towards increasingly personalized patient management. Clinical evaluation and attention to numerous extra-cardiac signs and symptoms are crucial in guiding the investigation and detection of CA in its subclinical phase. In this context, CPET can be highly useful for increasing clinical suspicion and non-invasively identifying early hemodynamic and metabolic abnormalities in CA patients, before the pathology becomes clinically apparent. In CA, CPET serves as an additional tool to detect early functional involvement of the disease. Furthermore, along with established risk assessment models, CPET is a valuable tool to optimize risk stratification in CA patients, offering a more comprehensive approach to functional capacity evaluation [68,109]. An additional challenge lies in identifying early clinical worsening in patients with a known CA diagnosis. CPET, having been used in therapy response evaluation, can also fulfill this role. Future clinical studies investigating CPET’s capability to assess response over follow-up periods with all the currently available pharmacological categories, including those that are still emerging, will be crucial. It will be especially important to confirm CPET’s role as an integrated part of examinations to identify signs of early clinical deterioration in individual patients, with the aim of optimizing therapy and achieving a “tailored clinical management” for patients affected by CA.

## 9. Conclusions

The utilization of CPET in the management of patients with cardiac amyloidosis, including both ATTR and AL types, is paramount in assessing disease progression, determining prognostic outcomes, and guiding therapeutic interventions. By objectively measuring cardio-respiratory performance and key parameters such as VO_2_ peak and exercise capacity, clinicians can tailor treatment approaches to individual patient needs, ultimately enhancing personalized care and improving clinical outcomes. CPET serves as a valuable tool in tracking disease trajectory, evaluating the efficacy of disease-modifying therapies, and making informed decisions regarding treatment initiation and adjustment. The integration of CPET into patient management strategies represents a significant advancement in personalized medicine approaches for cardiac amyloidosis, with the ultimate goal of enhancing the quality of life and overall prognosis for these challenging patient populations.

## Data Availability

No new data were created or analyzed in this study.
